# Protection of UVB-Induced Photoaging by Fuzhuan-Brick Tea Aqueous Extract via MAPKs/*Nrf2*-Mediated Down-Regulation of MMP-1

**DOI:** 10.3390/nu11010060

**Published:** 2018-12-28

**Authors:** Peijun Zhao, Md Badrul Alam, Sang-Han Lee

**Affiliations:** 1 Department of Food Science and Biotechnology, Graduate School, Kyungpook National University, Daegu 41566, Korea; laputaily@hotmail.com (P.Z.); mbalam@knu.ac.kr (M.B.A.); 2 Food and Bio-Industry Research Institute, Inner Beauty/Antiaging Center, Kyungpook National University, Daegu 41566, Korea

**Keywords:** anti-oxidant, anti-photoaging, heme oxygenase-1, nuclear factor erythroid 2-related factor 2 (*Nrf2*), matrix metalloproteinase-1 (MMP-1)

## Abstract

Ultraviolet B (UVB) irradiation is viewed as the principal inducer of skin photo-aging, associated with acceleration of collagen degradation and upregulation of matrix metalloproteinases (MMPs). The ethnic groups of southern/western China use Fuzhuan brick-tea (FBT) as a beverage and as a nutritional supplement. In this study, we scrutinized the antagonistic effects of aqueous extract of Fuzhuan-brick tea (FBTA) on skin photo-aging in UVB-exposed human keratinocyte (HaCaT) cells. FBTA exhibited strong antioxidant activity and quenched UVB-induced generation of cellular reactive oxygen species (ROS) without showing any toxicity. FBTA was capable of combating oxidative stress by augmenting messenger RNA (mRNA) and protein levels of both phase I and phase II detoxifying enzymes, especially heme oxygenase 1 (HO-1), by upregulating the nuclear factor erythroid 2-related factor 2 (*Nrf2*)-mediated pathway in HaCaT cells via the phosphorylation of p38 and extracellular signal-regulated kinase (ERK). FBTA also downregulated the expression of matrix metalloproteinase-1 (MMP-1) while upregulating type I procollagen by modulating *Nrf2* signaling in UVB-irradiated HaCaT cells. Collectively, our results show that FBTA might be useful as a functional food while being a good candidate in the development of cosmetic products and medicines for the remedy of UVB-induced skin photo-aging.

## 1. Introduction

Ultraviolet (UV) irradiation is viewed as one of the main factors causing structural and functional alterations in the skin, triggering skin aging [1]. Accumulating evidences show that skin photo-aging induced by UV-irradiation is associated with either excessive production of reactive oxygen species (ROS) or inflammatory mediators and disturbance of extracellular matrix (ECM) proteins [2,3]. In particular, UVB-stimulated redundant formation of intracellular ROS can cause an imbalance of cellular oxygen levels, triggering oxidative stress and impairing the antioxidant defense system, causing of photo-aging [4]. ROS also boost the production of matrix metalloproteinases (MMPs) which can enhance the degradation of ECM proteins such as collagen and elastin, which are the foremost structural proteins in skin connective tissue, thereby leading to skin photo-aging [5,6]. Therefore, stimulation of the endogenous antioxidant system and/or suppression of ROS regeneration might be an effectual approach to lessening UVB-stimulated photo-aging or skin damage.

Various phase I and phase II detoxifying enzymes are readily abundant in skin cells and are capable of quenching ROS, thereby sustaining cellular redox homeostasis [7,8]. Heme oxygenase 1 (HO-1), among these antioxidant proteins, plays a pivotal role in protecting ROS-induced oxidative stress-mediated skin damage. [9]. It is noteworthy that the activation of nuclear factor E2-related factor 2 (*Nrf2*) is crucial for the upregulation of HO-1. Under quiescence, *Nrf2* is dormant in the cytoplasm due to the Kelch-like ECH-associated protein 1 (Keap1). However, responses to oxidative stress or conformational changes of Keap1 by inducers facilitate the nuclear translocation of *Nrf2* and binding to antioxidant response elements (ARE), modulating the expression of various antioxidant enzymes and mitigating ROS generation [10,11,12]. Furthermore, UV-induced oxidative stress has been shown to modulate the phosphorylation of mitogen-activated protein kinases (MAPKs) and induce MMP secretion and collagen destruction [13]. It is well known that enhanced MMP-1 secretion as well as suppression of type I procollagen by UV-irradiation are the most distinguished features of photoaged skin [14]. Thus, agents with potential antioxidant properties that lessen MMP-1 production and accelerate procollagen type I synthesis are deemed as potential nominees for prevention of skin photoaging.

**Figure 1:**
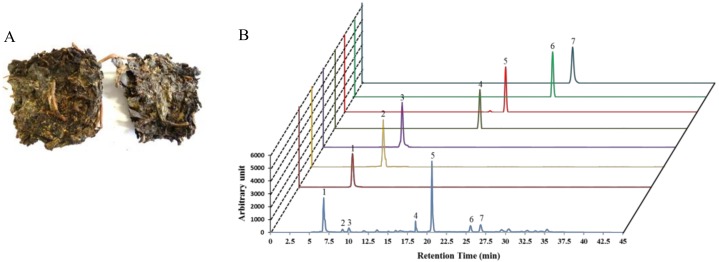
Fuzhuan-brick tea (FBT) (**A**) with the “golden flora” (the yellow dots) in its leaves. (**B**) High pressure liquid chromatography (HPLC) profile of Fuzhuan-brick tea aqueous extract (FBTA) with standards, including gallic acid (peak 1), theaflavins (peak 2), theobromine (peak 3), epigallocatechin (EGC) (peak 4), caffeine (peak 5), epicatechin (EC) (peak 6), and epigallocatechingallate (EGCG) (peak 7). A serving of 100 μg/mL of FBTA solution contains ~10 μM gallic acid, ~2 μM theoflavins, ~2 μM theobromine, ~4 μM EGC, ~15 μM caffiene, ~2 μM EC, and ~2 μM EGCG.

Tea (*Camellia sinensis*) is one of the most extensively consumed beverages worldwide and is comprehensively associated with numerous biological functions. Teas such as unfermented (green tea), semifermented (oolong tea), and fermented tea or black tea (Fuzhuan-brick tea, pu-erh tea, or liubao tea) are extensively dependent on the degree of fermentation and the production process. Among them, Fuzhuan-brick tea (FBT), native to the Hunan province of China, is a popular beverage within ethnic groups in the border regions of southern/western China [15]. A unique fungal fermentation process with a mixture of several microorganisms (predominantly *Eurotium* spp.) controls the aroma, flavor, and the degree of quality of FBT, with a golden “fungal flora” appearing within the tea (Figure 1A) [16]. Mounting evidence has shown that the fermentation process results in FBT having a unique phytochemical profile, with teapolyphenol, theaflavins, and caffeine being dominant (Figure 1B, Supplementary Figure S1) compared to other types of tea [15,17,18,19]. Moreover, the aroma and taste of FBT are dependent on the presence of nitrogenous, carbonaceous, and volatile compounds [20]. Cumulative studies have reported that black teas possess various pharmacological activities such as lipid-lowering and anti-obesity [21], antioxidant [22], and anti-bacterial and anti-mutagenic [23] activities. However, no studies to date have been conducted to protect skin photoaging by black teas. We asked whether FBT is functionally affiliated with *Nrf2* and induces antioxidant enzymes, thereby hindering oxidative stress-mediated photo-aging. In the present study, emphasis was given to confirm the regulatory role of aqueous extract of FBT (FBTA) in the antioxidant capacity of HaCaT cells. We also elucidated the mechanism underlying oxidative stress-induced skin photo-aging by assessing the activation of *Nrf2* induced by FBTA.

## 2. Materials and Methods

### 2.1. Plant Materials and Extraction

The Fuzhuan brick tea (FBT) was purchased from the Hunan Yiyang Tea Factory (Hunan, Yiyang, China). The voucher specimens of the plant and extracts have been deposited in the Laboratory of Enzyme Biotechnology, Kyungpook National University, Daegu, Republic of Korea. After air dying, 100 g tea powder were mixed with 15-folds of distilled water (DW) and placed in a shaking incubator at 60 °C for 24 h. Then, the supernatant was collected with filter paper (No. 1 Whatman Schleicher Schuell, Keene, NH, USA), and dried using a rotary vacuum evaporator (Tokyo Rikakikai Co. Ltd., Tokyo, Japan). Finally, the aqueous extracts of FBT (FBTA) were subjected to lyophilization and dissolved in deionized water at a concentration of 30 mg/mL as a stock solution.

### 2.2. Radical-Scavenging Activity Assays

2,2-diphenyl-1-picrylhydrazyl (DPPH-) and 2,20-azino-bis(3-ethylbenzothiazoline-6-sulphonic acid (ABTS-) radical scavenging assays, a ferric reducing antioxidant power (FRAP) assay, a cupric-reducing antioxidant capacity (CUPRAC) assay, and an oxygen radical absorbance capacity (ORAC) assay were carried out to evaluate the hydrogen and electron-donating capacity of FBTA, by which we confirmed the cell-free antioxidant potentiality of FBTA using previously described methods [24].

### 2.3. Cell Culture, UVB-Irradiation and Cell Viability Assay

HaCaT cells (1 × 10^5^ cells/mL) were cultured in DMEM medium supplemented with fetal bovine serum (FBS) and penicillin/streptomycin at 37 °C in 5% CO_2_ incubator. Then, sub-confluent cells were treated with indicated concentration (f.c. (final concentration) 3, 10, 30, or 100 μg/mL) of FBTA for 24 h. Subsequently, the cells was exposed to UVB at a dose of 60 mJ/cm^2^ using a UVB source (Bio-Link Crosslinker, Vilber Lourmat, Cedex, France) set at a spectral peak of 312-nm for 20 s. After UVB irradiation, the cells were cultured in serum-free medium for 24 h. Cell viability was determined using the 3-(4,5-dimethylthiazol-2-yl)-2,5-diphenyltetrazolium bromide (MTT) colorimetric assay as described previously [11].

### 2.4. Measurement of Cellular ROS Generation

HaCaT cells (1 × 10^5^ cells/mL) were cultured with indicated concentration of FBTA (f.c. 10, 30, or 100 μg/mL) in 96-well black plates for 24 h and then exposed to UVB-irradiation (60 mJ/cm^2^), followed by a change in the media and further incubation for 24 h. After that, the cells were washed with PBS twice and treated with 25 μM 2’,7’-dichlorofluorescin diacetate (DCF-DA) for 30 min at 37 °C in a CO_2_ incubator. Finally, fluorescence intensity was measured at excitation and emission wavelengths of 485 and 528 nm, respectively, by a fluorescence microplate reader (Victor3, PerkinElmer, Waltham, MA, USA).

### 2.5. Reverse Transcription-Polymerase Chain Reaction (RT-PCR) 

HaCaT cells (1 × 10^5^ cells/mL) were cultured with indicated concentration of FBTA (f.c. 10, 30, or 100 μg/mL) in 6-well plates for 24 h. TRIzol reagent (Life Technologies, Gaithersburg, MD, USA) was used for the extraction of total RNA and complementary DNA (cDNA) was prepared using RT & GO Mastermix (MP Biomedicals, Seoul, Republic of Korea) and served as the PCR template. A PCR Thermal Cycler Dice TP600 (Takara Bio Inc., Otsu, Japan) was used to carry out RT-PCR using the various primer sequences (Supplementary Data Table S1) [24,25]. After electrophoresis, ethidium bromide staining was performed to visualize the PCR products. 

### 2.6. Cell Lysates and Western Blotting

The lysates of HaCaT cells were prepared using radioimmunoprecipitation assay (RIPA) buffer with a phosphatase and protease inhibitor cocktail (Sigma-Aldrich, St. Louis, MO, USA) and the bicinchoninic acid (BCA) method was applied to quantify the protein content. A nuclear/cytosolic fractionation kit (Sigma-Aldrich, St. Louis, MO, USA) was used for the extraction of nuclear proteins. Aliquots of 50 μg of total proteins were used to carry out the Western blot analysis using various antibody (Supplementary Data Table S2) according to our previously described methods [24,25]. 

### 2.7. Statistical Analysis

The data were expressed as the mean ± standard deviation (SD; *n* = 3) and analyzed using the GraphPad Prism Software (GraphPad Software, Inc., San Diego, CA, USA). Statistical analysis was performed using one-way analysis of variance (ANOVA), followed by Dennett’s test. A value of *p* < 0.05 was considered as significant.

## 3. Results

### 3.1. Radical Scavenging Abilities

**Figure 2:**
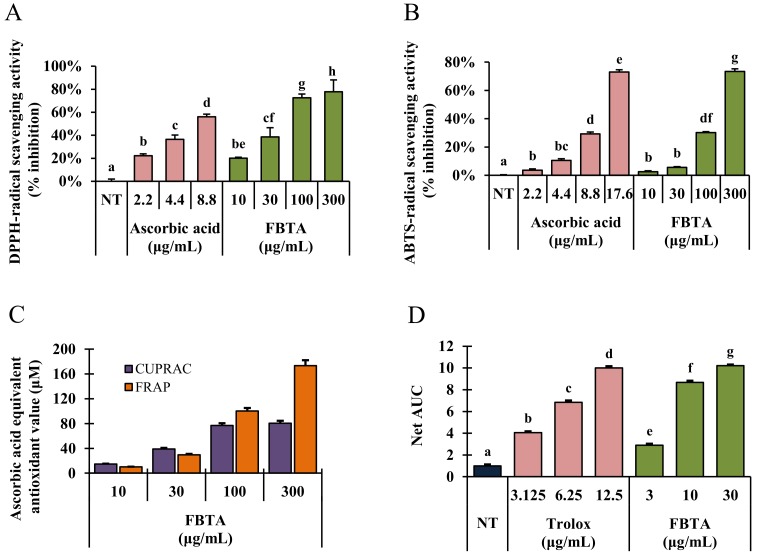
Cell free antioxidant activity of FBTA. (**A**) 2,2-diphenyl-1-picrylhydrazyl (DPPH-) and (**B**) 2,20-azino-bis(3-ethylbenzothiazoline-6-sulphonic acid) (ABTS)-radical scavenging activities. (**C**) Ferric- and (**D**) cupric-reducing activity was examined with different concentrations of FBTA in which ascorbic acid was used as standard. (**D**) The oxygen radical absorbance capacity (ORAC) activity of the samples was calculated by net area under the curve (net AUC). The different letters in each column are significant (*p* < 0.05). Different letters (a, b, c, d, e, f, g, be, cf, bc, df) are denoted as the statistical significance.

Various cell free antioxidant assay systems such as DPPH-, and ABTS-radical scavenging as well as FRAP, CUPRAC, and ORAC assays were carried out to determine the antioxidant ability of FBTA along with other commercially available dark teas such as pu-erh tea and liubao tea. As described in results, FBTA markedly scavenged DPPH-radicals by 79.85 ± 3.38% followed by pu-erh tea (79.25 ± 1.38%) and liubao tea (74.56 ± 3.08%) at a dose of 300 μg/mL (Figure 2A; Supplementary Figure S2). In ABTS-radical scavenging activity, FBTA showed the highest ABTS-radical scavenging activity (75.78 ± 2.25%), followed by liubao tea (71.35 ± 1.56%) and pu-erh tea (68.98 ± 2.45%) (Figure 2B, Supplementary Figure S2). Furthermore, in Figure 2C, FBTA expressed a strong reducing power ability with respect to CUPRAC and FRAP assays with ascorbic acid equivalent antioxidant value at 80.98 ± 1.25 μM and 162.52 ± 1.86 μM, respectively, at a dose of 300 μg/mL. On the other hand, pu-erh tea and liubao tea had 65.78 ± 0.95 μM and 81.53 ± 2.15 μM ascorbic acid equivalent antioxidant value, respectively, in the CUPRAC assay, as well as 125.64 ± 2.19 μM and 164.25 ± 3.21 μM ascorbic acid equivalent antioxidant value, respectively, in the FRAP assay (Supplementary Figure S3). FBTA also meaningfully and concentration-dependently raised the net area under the curve (AUC) value in ORAC assay, confirming its strong reducing power activity (Figure 2D). We also further evaluated the radical scavenging ability of the identified polyphenolics of FBTA, at their presumed concentration in FBTA. Interestingly, all the identified constituents exhibited potent radical scavenging activity in the order of gallic acid > caffeine > (EGCG) > (EGC); > (EC) ≅ theaflavins > theobromine (Supplementary Figure S4).

### 3.2. Assay of Cell Viability in UVB-Irradiated HaCaT Cells

**Figure 3:**
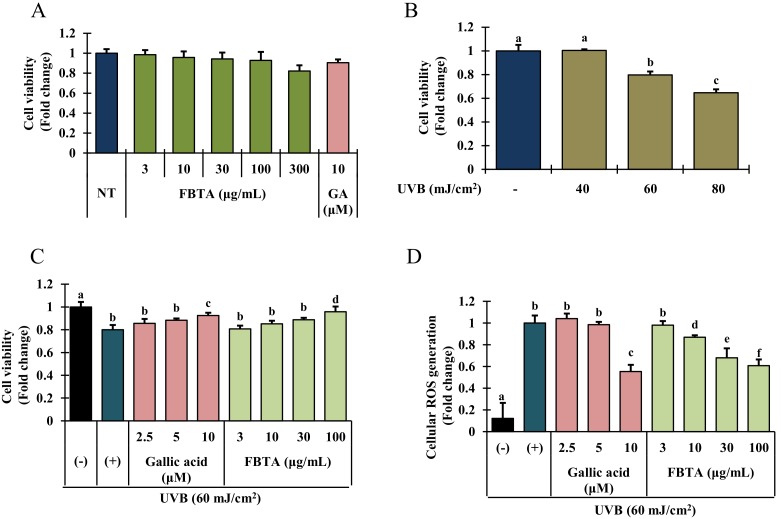
Cell viability activity of FBTA was evaluated by 3-(4,5-dimethylthiazol-2-yl)-2,5-diphenyl tetrazolium bromide (MTT) assay. (**A**) HaCaT cells (1 × 10^5^) were treated with FBTA (f.c. (final concentration) 3–300 μg/mL) and gallic acid (10 μM) for 24 h; (**B**) HaCaT cells were seeded (1 × 10^5^ cells/mL) in 96-well plates for 24 h, and then irradiated with UVB (40, 60 and 80 mJ/cm^2^) followed by incubation for 24 h; (**C**) Cells (1 × 10^5^) were pretreated with FBTA (10, 30 and 100 μg/mL) and gallic acid (GA) (2.5, 5, and 10 μM) for 24 h and then irradiated with UVB (60 mJ/cm^2^). The cell viability was measured by MTT assay as described in materials and methods. The different letters of each column show significance (*p* < 0.05). (**D**) Pretreated HaCaT cells by FBTA and gallic acid were exposed to UVB irradiation (60 mJ/cm^2^). Reactive oxygen species (ROS) levels were determined according to the Materials and Methods section. The different letters of each column show significance (*p* < 0.05). Different letters (a, b, c, d, e, f) are denoted as the statistical significance.

To examine the cytotoxic effects of UVB and FBTA on HaCaT cells, an MTT assay was performed. Since gallic acid at its putative concentration (9~10 μM) in FBTA has shown the highest antioxidant effects in cell free in vitro antioxidant assays, we used gallic acid as a positive control for cell-based assays. Gallic acid (f.c. 10 μM) and FBTA (f.c. 3 to 300 μg/mL) treatment did not show any significant cytotoxicity for 24 h (Figure 3A). Thus we fixed the concentration of FBTA as 3–100 μg/mL for further cell-based experiments. As shown in Figure 3B, UVB-irradiation significantly suppresses the cell growth in a concentration-dependent fashion. Interestingly, FBTA and gallic acid treatment substantially protected the cells from the toxic effect of UVB-irradiation at dose of 100 μg/mL and 10 μM, respectively (Figure 3C).

### 3.3. Effects of FBTA on ROS Generation

Spectrofluorometric analysis disclosed that UVB exposure significantly increased the intracellular ROS production in HaCaT cells (Figure 3D, column 2), whereas FBTA treatment significantly and dose-dependently repressed this trend (Figure 3D, column 6 to 9). In addition, to investigate the major constituents among the identified molecules in FBTA, which plays the crucial role in anti-photoaging effects of FBTA, we also examined the antagonist effect of all identified molecules, at their putative concentration in FBTA, on UVB-induced cellular ROS production. Our results revealed that gallic acid exhibited the highest quenching effects on cellular ROS generation, suggesting that gallic acid might be a principal constituent of FBTA for exhibiting anti-photoaging effects ( Data Supplementary Data Figure S5) through lessening oxidative stress.

### 3.4. Effects of FBTA on Phase I and Phase II Antioxidant Enzyme Expression in HaCaT Cells

**Figure 4:**
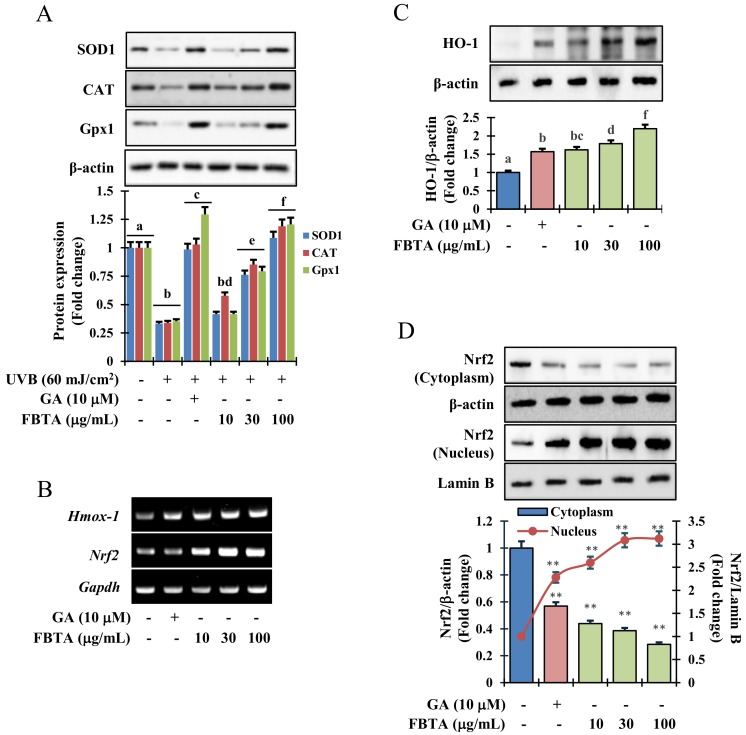
Effects of FBTA on the antioxidant enzyme expression through the nuclear factor erythroid 2-related factor 2 (*Nrf2*) signaling pathway. (**A**) FBTA pretreated HaCaT cells were exposed with UVB (60 mJ/cm^2^), and the protein expression of superoxide dismutase 1 (SOD1), catalase (CAT), and glutathione peroxidase 1 (GPx-1) was detected by immunoblotting. The different letters of each column indicate significance (*p* < 0.05); (**B**) After treatment of HaCaT cells by FBTA, messenger RNA (mRNA) expressions of *Hmox-1* and *Nrf2* were detected by RT-PCR. (**C**,**D**) Heme oxygenase 1 (HO-1) and *Nrf2* expressions were detected by immunoblotting. Densitometric analysis was carried out to quantify the band intensity by β-actin normalization. ** *p* < 0.01 compared to the normal cells. GA: gallic acid. Different letters (a, b, c, d, e, f, g, bc, bd) are denoted as the statistical significance.

Results of immunoblotting analysis revealed that UVB-irradiation dramatically lessened the protein expression of phase I antioxidant enzymes such as superoxide dismutase 1 (SOD1), catalase (CAT), and glutathione peroxidase 1 (GPx-1). Interestingly, FBTA and gallic acid treatment expressively upregulated the protein levels in a dose-dependent manner (Figure 4A). Likewise, the transcriptional and translational level of HO-1, one of the phase II detoxifying enzymes, was also boosted by FBTA in concentration-dependent fashions (Figure 4B,C, Supplementary Figure S6). The information advocates an antioxidant role of FBTA through acceleration of the expression of antioxidant enzymes.

### 3.5. Acceleration of HO-1 Enzymes via Nrf2 Nuclear Translocation in HaCaT Cells

**Figure 5:**
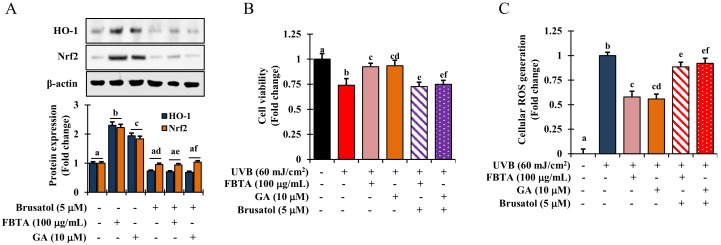
FBTA protects cell death by quenching cellular ROS through activation of *Nrf2*. Brusatol (f.c 5 μM) was added to the HaCaT cells and incubated for 30 min prior FBTA and gallic acid treatment. (**A**) HO-1 and *Nrf2* protein expression was detected by immunoblotting. Densitometric analysis was carried out to quantify the band intensity by β-actin normalization. The different letters of each column is significant (*p* < 0.05). GA: gallic acid; (**B**) Cells (1 × 10^5^) were treated with brusatol for 30 min, followed by treatment with FBTA (f.c 100 μg/mL) and gallic acid (10 μM) for 24 h and were then subjected to UVB (60 mJ/cm^2^) insult; (**B**) Cell viability was measured by MTT assay; (**C**) Cellular ROS generation was determined. The different letters of each column is significant (*p* < 0.05). Different letters (a, b, ad, ae, af, cd, ef) are denoted as the statistical significance.

Mounting evidence suggests that the redox sensitive transcription factor *Nrf2* inevitably harmonizes the cellular antioxidant function by the triggering of a series of antioxidant genes, thereby acting against photo-aging in the skin [8]. We hypothesized that the effects FBTA against photo-aging could be due to its persuasive antioxidant capacity. To validate this, we determined the profile of mRNA and nuclear translocation of *Nrf2* in FBTA-treated HaCaT cells. As shown in Figure 4B, the transcriptional level of *Nrf2* was steadily raised in FBTA- and gallic acid-treated HaCaTs. Likewise, immunoblotting analysis revealed that FBTA and gallic acid enhanced the nuclear translocation of *Nrf2*, while simultaneously lessening the cytosolic *Nrf2* level (Figure 4D). Next, to authenticate the *Nrf2*-induced HO-1 expression by FBTA, we treated the cells by brusatol (f.c. 5 μM), a specific inhibitor of *Nrf2*, before FBTA and gallic acid treatment. As expected, brusatol significantly suppressed *Nrf2* expression, and reserved the FBTA as well as gallic acid effects (Figure 5A). In addition, the induction of HO-1 protein by FBTA and gallic acid was also effectively terminated at brusatol-treated cells (Figure 5A). These findings proposed that FBTA can improve the antioxidant defense system via upregulation of *Nrf2*-mediated HO-1 expression. Then, we sought to define whether FBTA could suppress oxidative cell death through the activation of *Nrf2* signaling. Remarkably, the cell proliferation and scavenging of ROS by FBTA was partially reduced in the presence of *Nrf2* inhibitors (Figure 5B,C), signifying that the activation of *Nrf2* signaling by FBTA is involved in the protection of UVB-stimulated oxidative stress-induced cell death.

### 3.6. Effects of FBTA on the MAPK Signaling Pathway

**Figure 6:**
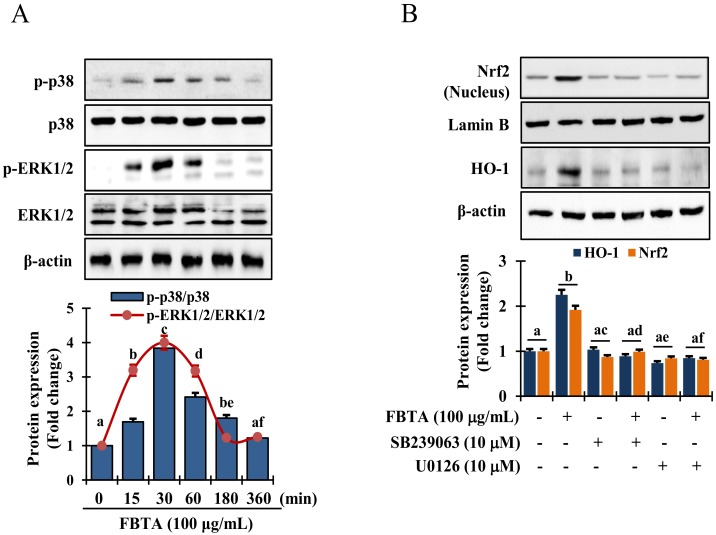
FBTA activates the mitogen-activated protein kinases (MAPKs) signaling pathway. (**A**) HaCaTs were treated with FBTA (100 μg/mL) at the indicated time point and the activated and non-activated forms of extracellular signal-regulated kinase 1 and 2 (ERK 1/2), and p38 were identified by immunoblotting assay. The different letters of each column indicate significance (*p* < 0.05). (**B**) Cells were treated with specific inhibitor U0126 and SB239063 in the presence and absence of FBTA (f.c. 100 μg/mL). *Nrf2* and HO-1 expressions were analyzed by immunoblotting. Densitometric analysis was carried out to quantify the band intensity by β-actin normalization. The different letters of each column indicate significance (*p* < 0.05). Different letters (a, b, c, d, ac, ad, ae, af, be) are denoted as the statistical significance.

It has been reported that activation of MAPKs act as a crucial upstream signaling in modulating the activation of *Nrf2* [24]. Thus, to reveal the mechanics responsible for *Nrf2* activation, cells were pretreated with FBTA for indicated time interval and immunoblotting assay was performed to assess the phosphorylation of p38 mitogen-activated protein kinase, and extracellular signal-regulated kinase 1 and 2 (ERK1/2). Interestingly, FBTA treatment substantially augmented the phosphorylation of p38 and ERK1/2 after 30 min (Figure 6A). However, there was no detectable c-Jun N-terminal kinase (JNK) phosphorylation in FBTA-treated HaCaT cells (Supplementary Figure S7). Thus, to confirm whether FBTA-modulated *Nrf2*-induced HO-1 upregulation is associated with the MAPK signaling cascade, cells were treated with specific p38 and ERK1/2 inhibitors, such as SB239063 and U0126, respectively, before being treated with FBTA. FBTA exhibited the potential to accrue the protein expression of *Nrf2* and HO-1, while p38 and ERK1/2 inhibition intensely reversed this trend (Figure 6B). These data acknowledge that ERK and p38 are required in FBTA-induced triggering of *Nrf2*-mediated HO-1 expression in in HaCaT cells.

### 3.7. Effects of FBTA on the Expressions of MMP-1 and Procollagen Type I

**Figure 7:**
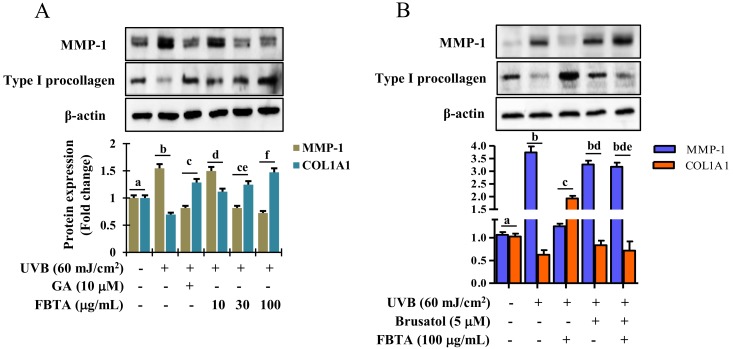
Effects of FBTA on matrix metalloproteinases (MMP)-1 and type I procollagen expression in UVB-stimulated HaCaT cells. The cells were pretreated with FBTA for 24 h, followed by UVB-irradiation. (**A**) MMP-1 and type I procollagen proteins were quantified by immunoblotting. The different letters of each column indicate significance (*p* < 0.05); (**B**) Cells were treated with brusatol for 30 min, followed by FBTA treatment (f.c. 100 μg/mL) for 24 h and were then subjected to UVB insult; the expressions of MMP-1 and type I procollagen were then analyzed by immunoblotting. Densitometric analysis was carried out to quantify the band intensity by β-actin normalization. The different letters of each column indicate significance (*p* < 0.05). Different letters (a, b, c, d, f, ce, bd, bde) are denoted as the statistical significance.

Accumulating research addressed that profound generation of MMPs and debasement of type I procollagen by UVB-irradiation predominantly leads to the pathogenesis of skin photoaging [14,26]. Thus to examine whether FBTA protects skin photoaging, UVB-exposed HaCaT cells were treated with FBTA and gallic acid and the expression of MMP-1 and type I procollagen was measured by Western blotting. Results displayed that FBTA treatment significantly downregulated the UVB-induced overexpression of MMP-1, as does gallic acid (Figure 7A). UVB alone induced a salient debasement of type I procollagen in HaCaT cells, while FBTA and gallic acid amended this trend. Nevertheless, FBTA and gallic acid remarkably elevated type 1 procollagen levels in UVB-stimulated cells (Figure 7A). These findings suggest that FBTA could prevent UVB-induced photoaging by lessening the MMP-1 upregulation and type I procollagen downregulation in skin keratinocytes, probably due to the presence of gallic acid, because during the permeation process, galloyl-catechins are metabolized by skin esterase and produce more gallic acid in the skin [27]. It is noteworthy that *Nrf2* plays a favorable role in delaying skin photoaging via the regulation of MMPs and type I procollagen [28,29]. There is a furthering pharmacological approach to delay skin photoaging by natural products via modulating *Nrf2*-induced antioxidant defense to combat oxidative stress. Thus, we examined *Nrf2* inhibition studies using brusatol in order to confirm the role of *Nrf2* activation in FBTA-mediated anti-photoaging effects against UVB exposure. Our results demonstrated that UVB-induced upregulation of MMP-1 in brusatol-treated cells remained high even after FBTA treatment, while FBTA extensively inhibited the MMP-1 expression (Figure 7B). Furthermore, FBTA treatment did not restore the UVB-induced degradation of type I procollagen in *Nrf2* inhibited cells (Figure 7B). These finding clarify that FBTA failed to hinder the UVB-induced MMP-1 overexpression and downregulation of type I procollagen in the absence of *Nrf2*.

## 4. Discussion

Epidemiological studies addressed the number of photoaged skin patients are increasing due to overexposure of solar UV-irradiation. Chronic exposure of skin to solar UV radiation causes oxidative stress, ROS-mediated DNA damage, and modulation of extracellular matrix (ECM) components such as MMPs and collagen, thereby hastening skin photo-aging [4]. Among the cells of epidermis, keratinocytes are dominate and can absorb UVB radiation. UVB-induced photo-toxicity (photo-aging) to keratinocyte was characterized by a decline of cell viability (Figure 3A). Pretreatment of FBTA and gallic acid lessened the photo-toxicity triggered by UVB exposure, thereby protecting keratinocyte cells against UVB irradiation. Various concentrations of FBT (50–200 μg/mL) did not exhibit cytotoxicity towards Caco-2 cells and also protected the cells against H_2_O_2_-induced oxidative stress [30]. Our results are supported by previous findings that, FBTA had dermato-protective properties against UVB irradiation.

It is well known that elderly people have lower endogenous antioxidants activity resulting in more vulnerable to UV-irradiated skin damage, thus the improved strategies for skin photoprotection are needed. Botanicals with antioxidant properties are viewed as potential therapeutic agents to treat skin disorders such as photo-aging [31]. FBTA showed strong hydrogen- as well as electron-donating capacity in various in vitro cell free antioxidant assay systems (Figure 2), thereby confirming that FBTA has very strong antioxidant activity. In addition, we compared total phenol and flavonoid content (Supplementary Figure S1) as well as antioxidant activity with other commercially available dark teas including pu-erh tea and liubao tea (Supplementary Figures S2–S4). FBTA had strong ABTS-radical scavenging potential as compared to pu-erh tea and liubao tea, while in the DPPH-radical scavenging assay, FBTA and pu-erh tea showed similar effects. In contrast, liubao tea showed highest reducing power activity as compared to FBTA and pu-erh tea in both the CUPRAC and FRAP assay system. Our results are also supported by a previous study which stated that the polyphenolics and antioxidant activities of dark tea are dependent on the degree of fermentation. Excessive pile-fermentation reduced the antioxidant activities of dark tea [32,33].

The UVB irradiation of the epidermis causes ROS generation, resulting in attenuation of SOD, CAT, and GPx1 activity and hastening of oxidative damage [34]. Here, we found that FBTA treatment was markedly reserved the UVB-induced ROS generation (Figure 3D). In addition, among the identified bioactive molecules of FBTA, gallic acid showed the highest cellular ROS quenching activity (Supplementary Figure S5), suggesting that gallic acid could play the major role in attenuating the oxidative stress by FBTA. Moreover, FBTA treatment also restored the endogenous antioxidant enzymes such as SOD1, CAT, and GPx-1 in UVB-exposed HaCaT cells (Figure 4A). Our results also supported by the previous studies which reported the FBT significantly protected high fat diet-induced oxidative stress in liver by ameliorating the levels of SOD, CAT, and GSH-Px [35].

Heme oxygenase-1 (HO-1), a phase II-detoxifying enzymes, can convert heme into bilirubin, which acts as a strong antioxidant capable of protecting against cell death from oxidative insult [24]. Besides, a consistent increase in oxidative stress mediated by ROS results in a lowering the cellular HO-1 levels [36]. Upon treatment, FBTA showed a substantial enhance in both transcriptional and translational levles of HO-1 in HaCaT cells (Figure 4B,C, Supplementary Figure S2). Mounting evidence shows various polyphenols, such as gallic acid, EGC, and EGCG attenuate ROS-mediated oxidative stress-induced cell death through upregulation of HO-1 levels [37,38]. To validate this phenomenon, the transcriptional and translational level of *Nrf2*, the key regulator of HO-1 activation, was studied. Furthermore, Hirota et al. [39] discovered that knockdown of the *Nrf2* gene in mice exhibited the acceleration of UVB-induced photoaging process. Thus, *Nrf2*, a well-known redox-sensitive transcription factor, plays a critical role to protect cells against UVB-stimulated photoaging. The pharmacological approach for the activation of *Nrf2* has drawn substantial attention as a tactic for skin photoprotection [40]. Upon electrophilic and/or oxidative stress, *Nrf2* enters into the nucleus and triggers phase II-detoxifying enzymes such as HO-1, thereby indirectly ameliorating the cellular antioxidant defense system, and can protect skin against oxidative damage [41]. In our study, we found pretreatment of FBTA prompted *Nrf2* translocation of the nucleus, while inhibition of *Nrf2* strongly alleviated the upregulation of HO-1 (Figure 4D and Figure 5A), confirming that *Nrf2* regulates the expression of phase II antioxidant enzymes such as HO-1. Gallic acid activates the *Nrf2*-mediated induction of HO-1 and glutathione-s-transferase alpha 3, preventing liver injury [42]. The apocarotenoid bixin, a natural food additive, was revealed to activate *Nrf2* and prevent skin damage by solar UV irradiation [40]. FBTA causes the activation of *Nrf2* and boosts antioxidant capacity, subsequently lessening UVB-induced oxidative stress by suppressing ROS generation. This suggests that FBTA acts as an *Nrf2* activator and has protective properties against photooxidative stress through the activation of *Nrf2*. Cumulating evidence has shown that the activation of *Nrf2* by various cytoprotective phytochemicals are involved in the modulation of various signaling molecules such as MAPKs including ERK1/2, p38, and JNK [8,43]. Our results demonstrated that FBTA-mediated *Nrf2* activation is accomplished through ERK1/2 and p38 MAPK signaling in HaCaT cells (Figure 6A). Pharmacological inhibition of these signaling cascades abolished FBTA-induced *Nrf2* nuclear accumulation and subsequently inhibited HO-1 amplification (Figure 6B). A current study disclosed that p38 and ERK1/2 are crucial for *Nrf2*-mediated HO-1 augmentation in HSC-3 cells [43]. Gallic acid activated the p38 pathway, enhancing the accumulation of *Nrf2* into nucleus and modulation of phase II P-form of phenol sulfotransferase, resulting in protecting oxidative stress induced HepG2 cell death [44]. Based on our findings, we speculated that p38 and ERK1/2 signaling molecules plays a pivotal role in *Nrf2* activation and demonstrate the dermato-protective properties of FBTA.

UVB-induced ROS were reported to be associated with MMP production and modulation of collagen and elastin components of ECM, thereby causing photo-aging and skin damage [7]. Therefore, natural products and/or nutrients with antioxidative properties which can suppress ROS production mitigate the upregulation of MMPs while enhancing type I procollagen synthesis are thought to be novel approaches to protecting against photo-aging. Dietary *Foeniculum vulgare* Mill extract attenuated UVB-induced skin photo-aging by suppression of ROS production and expression of MMP-1, while increasing the type I procollagen level in hairless mice [45]. Likewise, pretreatment with youngiasides A and C, from *Youngia denticulatum*, taken as a wild vegetables, has been reported to act as an antioxidant, abolishing UVB-induced upregulation of MMP-1 and degradation of procollagen I in HaCaT cells [46]. Gallic acid exhibited protection of skin from UVB-induced photo-aging via negative modulation of MMP-1 secretion and positive regulation of type I procollagen in hairless mice [47]. Topical administration of spent coffee ground extracts downregulate of MMPs, thereby protecting skin from UVB-induced photo-aging in hairless mice [48]. Our results demonstrated that FBTA pretreatment mitigated UVB-induced MMP-1 upregulation in a dose-dependent fashion, and renovated type I procollagen in HaCaT cells (Figure 7A). Interestingly, this trend was blocked by the inhibition of *Nrf2* (Figure 7B). It is of note that type I procollagen biosynthesis is considerably diminished in photo-aged skin, causing a loss in dermal elasticity, while restoration of type I procollagen with FBTA is evidence of its dermato-protective effect. This anti photo-aging effect appears to be arbitrated via activation of *Nrf2*-mediated downregulation of MMP-1 in UVB-exposed HaCaT cells.

Unfermented (green tea), semifermented (oolong tea), and fermented (Fuzhuan-brick tea, pu-erh tea or liubao) teas are some of the most widely consumed beverages in the world. The major chemical constituents of teas are polyphenols. Among them, flavan-3-ol such as EGCG and ECG are dominant. Flavonoids, gallic acid, caffeine, and amino acid are also present. Interestingly, during the production of black tea, catechins such as EC, ECG, EGC, and EGCG are oxidized by polyphenol oxidase (PPO) and peroxidase (POD) and consequently dimerized to theaflavins and polymers (thearubigins). There are some studies reporting on the pharmacokinetics profile of tea polyphenols, while the pharmacokinetics profiles of black tea polyphenols, theaflavins, and thearubigins have not been studied extensively [49]. However, gallic acid metabolites such as 3-*O*-methylgallic acid and 4-*O*-methylgallic acid found in the urine of humans who took black tea and are considered as an index of black tea consumption [50]. Mounting evidence has revealed that the half-lives of tea polyphenols are 2–4 h in humans. After oral administration, the peak plasma concentration is in the low μM range, which can be achieved within 1–3 h. Generally, it is known that 2~4 cups/day of Fuzhuan brick tea are consumed by healthy Chinese people (70 kg). The plasma concentration of gallic acid was found to be 2.09 μmol/L after oral consumption of 200 mL (equivalent to 1 cup) of Assam black tea [51]. Thus, it may assumed that in a healthy volunteer who drinks 2~4 cup of black tea per day, the plasma concentration of gallic acid could equivalent be to 5~9 μmol/L; a gallic acid concentration of 10 μmol/L is physiologically effective for exhibiting anti-skin aging effects. A number of studies have stated the low μM concentrations of tea polyphenol have diverse biological effects such as anti-inflammatory, antioxidant, anti-proliferative, and photoprotective effects in vitro [49]. This is also supported in our current study, where the used concentrations of FBTA and gallic acid were in the μM range.

Further studies should be focused on (1) finding active ingredients contributing anti-skin aging potential by investigating synergistic effects of polyphenolics, small molecules, and/or undetected compounds in FBTA, and (2) the pharmacokinetic parameters of tea polyphenols after oral administration of FBTA in a mice model. These are rewarding in that the future data will be beneficial to consumers for better skin and inner beauty care.

## 5. Conclusions

**Figure 8:**
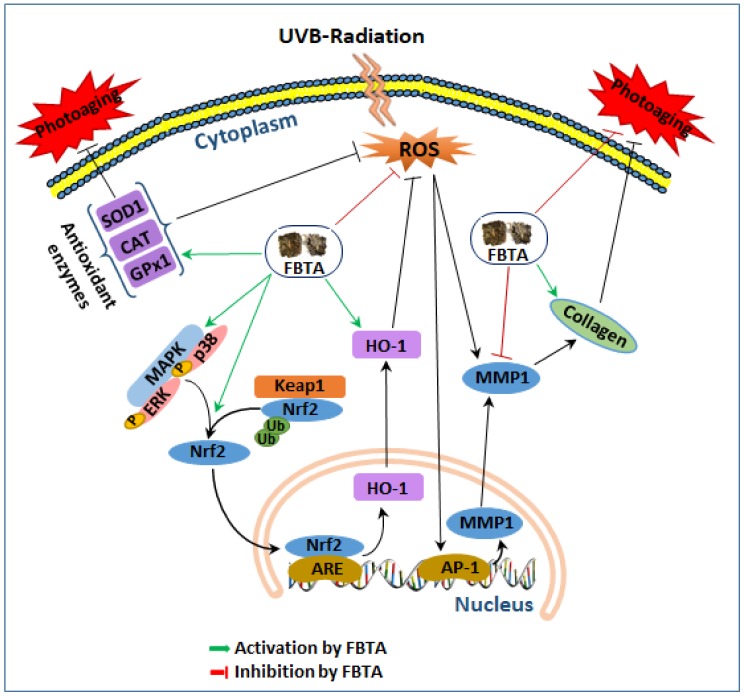
A proposed anti-aging mechanism of FBTA in the UVB-induced photoaging human keratinocytes. Ub, ubiquitin; ARE, antioxidant response element. Green arrows, activation by FBTA; red bars, inhibition by FBTA, black arrows, activation; black bar, inhibition.

Our findings revealed for the first time that FBTA pretreatment mitigated UVB-induced photoaging in human keratinocyte HaCaT cells (Figure 8). FBTA stimulated the nuclear translocation of *Nrf2* via induction of p38 and ERK1/2 phosphorylation and subsequently induced HO-1, thereby successively quenching UVB-induced ROS production. Most prominently, significant stimulation of MMP-1 and downregulation of type I procollagen by UVB was restored by FBTA in HaCaT cells, probably through the activation of *Nrf2*. Collectively, our results demonstrated molecular evidence that FBTA could inhibit UVB-induced photoaging via quenching of ROS and triggering of *Nrf2* signaling cascades.

## Supplementary Materials

Supplementary Table S1: List of the primers used in the study; Supplementary Table S2: List of the antibodies used in the study; Supplementary Figure S1: Total phenolic and flavonoid contents of the aqueous extract of Fuzhuan-brick tea, pu-erh tea, and libao tea; Supplementary Figure S2: DPPH- and ABTS-radical scavenging activities of the aqueous extract of Fuzhuan-brick tea, pu-erh tea, and libao tea; Supplementary Figure S3: Reducing power activities of the aqueous extract of Fuzhuan-brick tea, pu-erh tea, and libao tea in CUPRAC and FRAP assays; Supplementary Figure S4: DPPH-radical scavenging activities of the aqueous extract of Fuzhuan-brick tea (FBTA) and the identified constituents at their putative concentrations in FBTA; Supplementary Figure S5: Cellular ROS quenching effects of the aqueous extract of Fuzhuan-brick tea (FBTA) and the identified constituents at their putative concentration in FBTA; Supplementary Figure S6: Effect of FBTA on HO-1 and *Nrf2* expression in UVB-exposed HaCaT cells; Supplementary Figure S7: Effects of Fuzhuan-brick tea aqueous extract on the activation of JNK.
