# Supplementary material for: Protection of UVB-Induced Photoaging by Fuzhuan-Brick Tea Aqueous Extract via MAPKs/Nrf2-Mediated Down-Regulation of MMP-1
Source: Nutrients. 2018 Dec 28;11(1):60. doi: 10.3390/nu11010060 (PMC6357030; doi:10.3390/nu11010060)
Supplement: Supplementary file 1 [file nutrients-11-00060-s001.pdf]

SUPPLEMENTARY DATA SHEETS

# Protection of UVB-Induced Photoaging by Fuzhuan-Brick Tea Aqueous Extract via MAPKs/*Nrf2*-Mediated Down-Regulation of MMP-1

**Peijun Zhao [1,†], Md Badrul Alam [1,2,†] and Sang-Han Lee [1,2,*]**

[1]Department of Food Science and Biotechnology, Graduate School, Kyungpook National University, Daegu 41566, Republic of Korea
[2]Food and Bio-Industry Research Institute, Inner Beauty/Antiaging Center, Kyungpook National University, Daegu 41566, Republic of Korea
† These authors contributed equally to this work.

**Running head:** Attenuation of photo-aging by Fuzhuan-brick tea

**Corresponding authors:**

**Dr. Sang Han Lee;** E-mail: sang@knu.ac.kr

## 2. Materials and Methods

### *2.1. Drugs and chemicals*

2,2-Diphenyl-1-picrylhydrazyl (DPPH), neucaprine, 2,20-azino-bis(3-ethylbenzothiazoline-6-sulphonic acid) (ABTS), 2',7'-dichlorofluorescin diacetate (DCFH-DA), 3-(4,5-dimethylthiazol-2-yl)-2,5-diphenyltetrazolium bromide (MTT), and dimethylsulfoxide (DMSO) were purchased from Sigma Aldrich (St. Louis, MO, USA). Dulbecco's modified Eagle's medium (DMEM), fetal bovine serum (FBS), penicillin-streptomycin mixture (P/S), and 0.25% trypsin-EDTA were purchased from Gibco-BRL Life Technologies (Grand Island, NY, USA). All antibodies anti-SOD1, anti-CAT, anti-GPx-1, anti-HO-1, anti-MMP-1, anti-type I procollagen, and anti-*Nrf2* were purchased from Santa Cruz Biotechnology (Santa Cruz, CA, USA) as described in Table S2.

### *2.2. High-performance liquid chromatographic (HPLC) analysis*

Phytochemical characterization of FBTA, including standard molecules (gallic acid, theaflavins, theobromine, epigallocathecin, caffeine, epicatechin and epigallocatechingallate (EGCG)) was performed using an auto-sampler HPLC (HPLC-DAD) setup (Shimadzu, Japan) supported with a diode array detector (SPD-M20A) and LC solution software (ver. 1.22 SP1). A 5 μm-diameter particles packed C18 column (4.6 × 250 mm) was used for reverse-phase chromatographic analysis. The following gradient solvent system [acetonitrile (A) and 1% formic acid (B)] was employed with change in ratio at every time (min); solvent A (10%) was run up to 10 min, followed by 30, 50, 60, 90, and 20% for 15, 20, 25, 30, 35, and 40 min, respectively (λ = 280 nm). The injection volume was 20 μL, and the flow rate was maintained at 0.8 mL/min [1]. The phenolic components were identified based on the retention time compared with those of standard compounds.

**Table S2: List of the primer sets used in this study.**

| *Gene name* | | *Sequences* |
|---|---|---|
| *Hmox-1* | *forward* | *ACGCATATACCCGCTACCTG* |
| | *reverse* | *TCCTCTGTCAGCATCACCTG* |
| *Nrf-2* | *forward* | *ACATCCTTTGGAGGCAAGAC* |
| | *reverse* | *GGGAATGTCTCTGCCAAAAG* |
| *Gapdh* | *Forward* | *TTGTGATGGGTGTGAACCAC* |
| | *reverse* | *ACACATTGGGGGTAGGAACA* |

**Table S2: List of antibodies used in this study.**

| *Name* | *Catalog number* | *Company* | *Antigen* | *Host* |
|---|---|---|---|---|
| Anti-SOD1 | sc-101523 | Santa Cruz Biotechnology, Inc. | SOD1 | Mouse |
| Anti-CAT | sc-515782 | Santa Cruz Biotechnology, Inc. | CAT | Mouse |
| Anti-GPx-1 | sc-133160 | Santa Cruz Biotechnology, Inc. | GPx-1 | Mouse |
| Anti-HO-1 | sc-136256 | Santa Cruz Biotechnology, Inc. | HO-1 | Mouse |
| Anti *Nrf2* | sc-81342 | Santa Cruz Biotechnology, Inc. | *Nrf2* | Mouse |
| Anti-p-p38 | sc-166182 | Santa Cruz Biotechnology, Inc. | p38 | Mouse |
| Anti-p38 | BS3567 | Bioworld Technology, Inc. | p38 | Rabbit |
| Anti-p-ERK1/2 | sc-7383 | Santa Cruz Biotechnology, Inc. | ERK | Mouse |
| Anti-ERK1/2 | BS 6472 | Bioworld Technology, Inc. | ERK | Rabbit |
| Anti-p-JNK | BS 4322 | Bioworld Technology, Inc. | JNK | Rabbit |
| Anti-JNK | sc-7345 | Santa Cruz Biotechnology, Inc. | JNK | Mouse |
| Anti-MMP1 | sc-21731 | Santa Cruz Biotechnology, Inc. | MMP-1 | Mouse |

| | | | | |
|---|---|---|---|---|
| Anti-type I Procollagen | sc-376350 | Santa Cruz Biotechnology, Inc. | COL1A2 | Mouse |
| Anti-Lamin B | Sc-6217 | Santa Cruz Biotechnology, Inc. | Lamin B | Goat |
| Anti-β actin | Sc-47778 | Santa Cruz Biotechnology, Inc. | β-actin | Mouse |

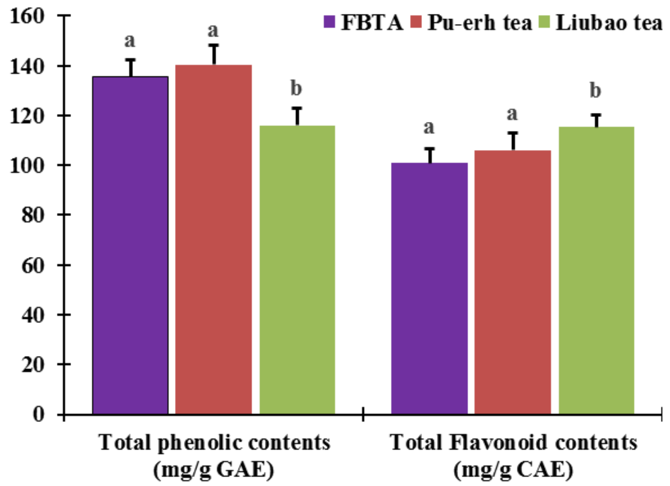


**Figure S1.** Total phenolic and flavonoid contents of the aqueous extract of Fuzhuan-brick tea, pu-erh tea and libao tea. The different letter in the same group indicate the difference between the teas is significant.

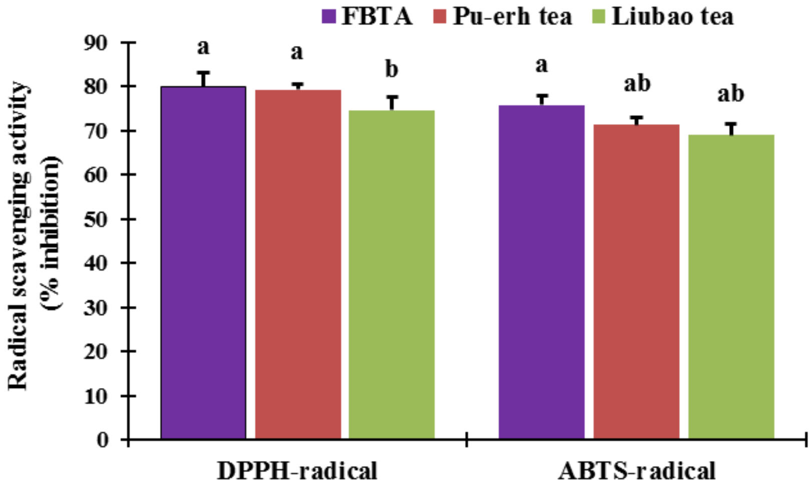


**Figure S2.** DPPH- and ABTS-radical scavenging activities of the aqueous extract of Fuzhuan-brick tea, pu-erh tea and libao tea. The different letter in the same group indicate the difference between the teas is significant.

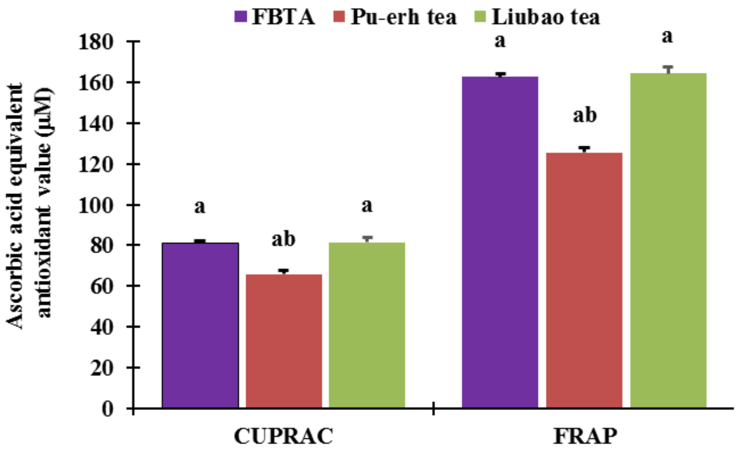


**Figure S3.** Reducing power activities of the aqueous extract of Fuzhuan-brick tea, pu-erh tea and libao tea in CUPRAC and FRAP assays. The different letters in the same group indicate the difference between the teas is significant.

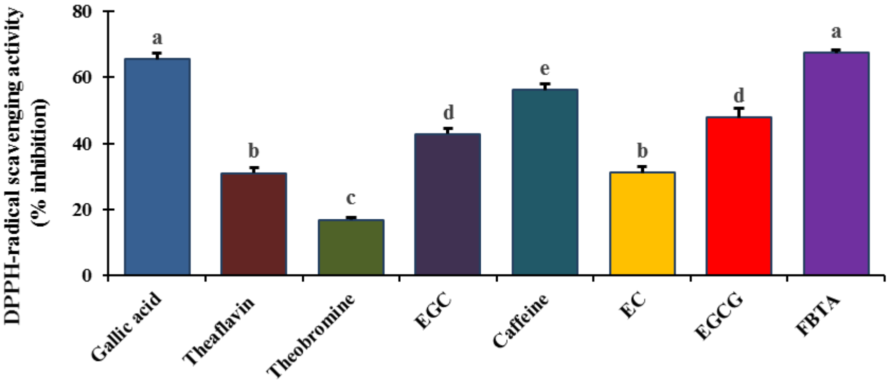


**Figure S4.** DPPH-radical scavenging activities of the aqueous extract of Fuzhuan-brick tea (FBTA) and the identified constituents at their putative concentrations in FBTA. The different letters indicate the difference between the constituents is significant ($p < 0.05$). The experimental concentration of gallic acid (10 μmol/L), theaflavins (2 μmol/L), theobromine (2 μmol/L), EGC (4 μmol/L), caffeine (15 μmol/L), EC (2 μmol/L), EGCG (2 μmol/L) and FBTA (100 μg/mL).

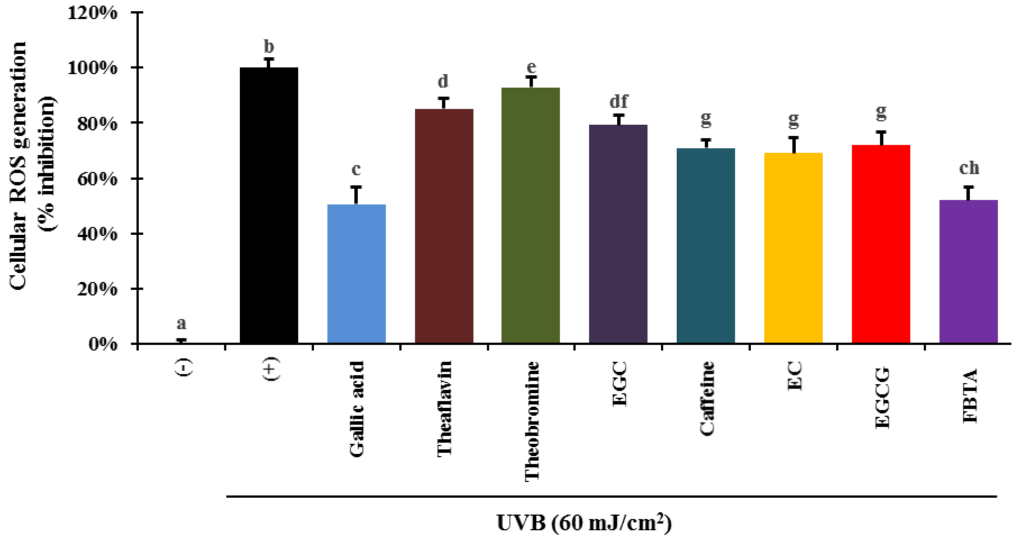


**Figure S5.** Cellular ROS quenching effects of the aqueous extract of Fuzhuan-brick tea (FBTA) and the identified constituents at their putative concentration in FBTA. The experimental concentration of gallic acid (10 μmol/L), theaflavins (2 μmol/L), theobromine (2 μmol/L), EGC (4 μmol/L), caffeine (15 μmol/L), EC (2 μmol/L), EGCG (2 μmol/L) and FBTA (100 μg/mL). Different letter of each column are significant ($p < 0.05$).

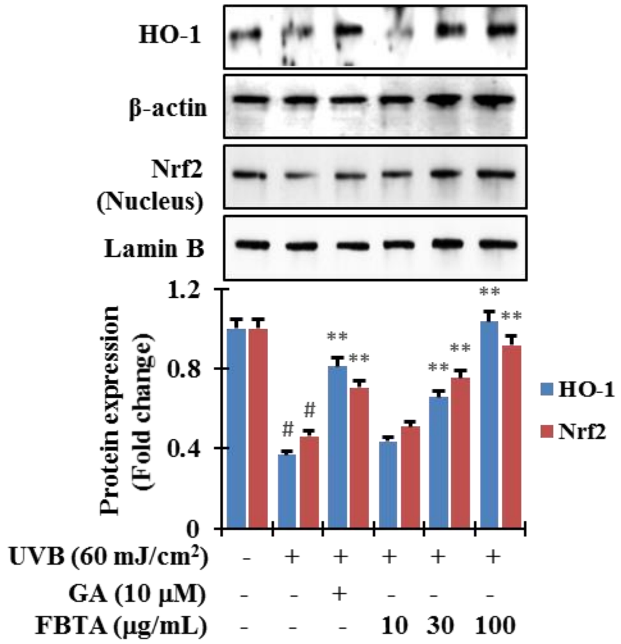


**Figure S6.** Effects of Fuzhuan-brick tea aqueous extract on *Nrf2* signaling. FBTA-pretreated HaCaT cells were exposed with UVB (60 mJ/cm$^2$), and the protein expressions of HO-1 and

*Nrf2* were detected by western blotting. #$p < 0.01$, compared to the normal cells, **$p < 0.05$, compared to UVB-irradiated cells.

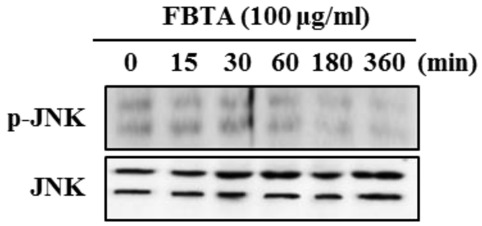


**Figure S7.** Effects of Fuzhuan-brick tea aqueous extract on the activation of JNK. HaCaT cells were treated with FBTA (100 µg/mL) for indicated time points and the activated and non-activated forms of JNK were identified by immunoblotting assay.

**References**

[1]. S.M. Brito, S.M.; Coutinho, H.D.; Talvani, A.; Coronel, C.; Barbosa, A.G.; Vega, C. Analysis of bioactivities and chemical composition of *Ziziphus joazeiro* Mart. using HPLC–DAD. Food Chem. **2015,** *186*, 185-191.
